# Consumption of Extruded Sorghum SC319 Improved Gut Microbiota at Genus Level and Reduced Anthropometric Markers in Men with Overweight: A Randomized Controlled Clinical Trial

**DOI:** 10.3390/nu15173786

**Published:** 2023-08-30

**Authors:** Haira Lúcio, Pamella Anunciação, Barbara da Silva, Alessandra da Silva, Valéria Queiroz, Carlos de Carvalho, Helena Pinheiro-Sant’Ana, Hercia Martino

**Affiliations:** 1 Nutrition and Health Department, Federal University of Viçosa, Campus Universitário, Av. Purdue, s/n, Viçosa 36570-900, MG, Brazil; haira.lucio@ufv.br (H.L.); nutripamella@gmail.com (P.A.); barbara.p.silva@ufv.br (B.d.S.); alessan.drasg94@gmail.com (A.d.S.); helena.santana@ufv.br (H.P.-S.); 2 Embrapa Milho e Sorgo, Rote MG 424, Km 65, Sete Lagoas 35701-970, MG, Brazil; valeria.vieira@embrapa.br; 3 Embrapa Agroindústria de Alimentos, Av. das Américas, nº 29.501, Guaratiba, Rio de Janeiro 23020-470, RJ, Brazil; carlos.piler@embrapa.br

**Keywords:** short-chain fatty acids, fecal pH, *Sorghum bicolor* L. Moench, weight loss, body fat, β diversity

## Abstract

Background: Sorghum is a cereal source of energy, carbohydrates, resistant starch, proanthocyanidins, and 3-deoxyanthocyanins; it promotes satiety by slowing digestion and benefits intestinal health. Objective: This study investigated the effects of extruded sorghum SC319 consumption on intestinal health, weight loss, and inflammatory markers in men with overweight. Methods: This was a randomized, controlled, single-blind clinical trial. Twenty-one men were randomly allocated into one of two groups: the sorghum group (test), which received 40 g of extruded SC319 whole sorghum (*n* = 10), or the wheat group (control), which received 38 g of extruded whole wheat (*n* = 11) for eight weeks. Results: The sorghum consumption increased the weight loss intragroup, decreased the body fat percentage intergroup, and did not change inflammatory markers, while the wheat group had increased IL-6 levels compared to baseline. Short-chain fatty acid production, fecal pH, and α and β diversity indexes did not differ intra- and intergroup after interventions. However, sorghum consumption decreased genus levels of *Clostridium_sensu_stricto 1*, *Dorea*, and *Odoribacter* and increased CAG-873 and *Turicibacter* compared to baseline. Further, sorghum showed a tendency (*p* = 0.07) to decrease the proteobacteria phyla compared to wheat. Conclusion: Extruded sorghum SC319 improved intestinal microbiota and body composition and promoted weight loss, demonstrating its prebiotic potential.

## 1. Introduction

Obesity is a disease related to complex interactions among genetic, socioeconomic, cultural, and environmental influences. It is a disease with multifactorial causes [1]. Obesity is frequently associated with the dysregulation of lipid, glucose, and cholesterol metabolism, in addition to increased oxidative stress and the establishment of low-grade chronic inflammation, which are risk factors for developing non-communicable chronic diseases [2,3]. It is estimated that the number of people with obesity is about to double by 2030, affecting one billion people worldwide [4].

The inflammatory environment caused by lipid accumulation results in a non-specific activation of the immune system, contributing to a large extent to the development of alterations in intestinal behavior, altering the functionality of enterocytes and other structures, and favoring the development of intestinal dysbiosis [5,6]. Intestinal health involves biological, mechanical, and structural changes that affect the intestinal environment. Thus, the main parameters related to intestinal health are the gut barrier, nutrient digestion and absorption, gut microbiome, fecal pH, SCFA production, mucus layer, barrier function, and mucosal immune responses [7,8]. The consequences of dysbiosis include the reduction in bacteria that synthesize short-chain fatty acids, the reduction in the activity of other bacteria and mucus production, and the increase in intestinal permeability [9]. Thus, these effects can increase the endotoxins in the circulation, activating the immune and inflammatory response [10,11].

Studies have shown that the intestinal microbiota can influence adiposity and weight gain [12], and weight gain can lead to changes in the intestinal microbiota composition [13]. In turn, weight loss interventions have been shown to induce changes in the gut microbiota. Emerging evidence suggests that the gut microbiota and its metabolites may play a pivotal role in mediating the effects of an energy restriction diet [14]. In addition, prebiotics acts as dietary fibers, serve as fuel for beneficial gut bacteria, and can also positively influence gut microbiota composition [15].

Sorghum grains (*Sorghum bicolor* (L.) Moench) are a source of resistant starch, energy, carbohydrates, proteins, and bioactive compounds, especially proanthocyanidins and 3-deoxyanthocyanins [16,17,18]. The SC319 sorghum genotype utilized in the present study is a genotype with high antioxidant capacity due to its chemical composition, which presents bioactive compounds such as phenolic acids, flavonoids, tannins, 3-deoxyanthocyanidins, and vitamin E [19,20,21]. Previous research of our group demonstrated that this sorghum genotype exhibits favorable palatability and consumer acceptance [21], and its consumption led to a notable reduction in the glycemic response of subsequent meals among healthy adults [22]. Studies in an experimental model have shown the benefits of consuming this grain on metabolic markers, including lipid and glucose metabolism [23,24,25]. In addition to the prebiotic effects of the dietary fiber present in whole grains such as sorghum, the SC319 sorghum genotype presents bioactive compounds such as proanthocyanidins and 3-deoxyanthocyanins, which may modulate the mucosal immune responses, inflammation, and the intestinal microbiota [26,27,28], thus promoting the intestinal health of individuals. Hence, it is essential to clarify the effect of extruded sorghum in the markers linked to the intestinal health of men with overweight, associating the consumption of this cereal with an energy restriction diet. This study aimed to investigate the potential beneficial effects promoted by extruded SC319 sorghum consumption on weight loss, inflammatory markers, and intestinal health, including fecal pH, SCFA production, target species, and gut microbiota composition in overweight men. As it is a genotype rich in tannins, this type of sorghum can favor weight and body fat loss and modulate the intestinal microbiota due to the reduced digestibility of macronutrients such as carbohydrates and proteins.

## 2. Materials and Methods

### 2.1. Raw Materials and Processing

Whole-grain sorghum (SC319 genotype) was grown in Nova Porteirinha, MG, Brazil, by Embrapa Milho e Sorgo. The grains were harvested in September 2013. They were milled into flour using a disc mill (Perten Instruments, Huddinge, Sweden) at position 2. The sorghum flour was combined with 10% fine granulated sugar (sucrose) and 0.5% iodized salt (NaCl). The mixture was processed using a twin-screw extruder (Clextral, Firminy, France) with a screw speed of 600 rpm and temperature ranging from 30 to 140 °C. The extruder had a screw diameter of 25 mm and a length of 1000 mm, resulting in an L/D ratio of 40. The die had four round openings measuring 2.0 mm in diameter and 9 mm in length. A gravimetric feeder (Schenck Process, Darmstadt, Germany) delivered the formulation to the extruder, while distilled water was added to adjust the moisture content to 12%. The extruded sorghum breakfast cereal was stored in polyethylene bags at 10 ± 2 °C.

Similarly, whole-grain wheat flour from SL Alimentos in Mauá da Serra, PR, Brazil, was processed with the addition of 10% sucrose and 0.5% iodized salt. The wheat flour mixture was extruded using a twin-screw extruder (Clextral, Firminy, France) at a screw speed of 200 rpm and temperature ranging from 50 to 143 °C. The processing conditions were comparable to those used for sorghum. The resulting whole-grain wheat breakfast cereal was also stored in polyethylene bags at 10 ± 2 °C until it was ready for consumption.

### 2.2. Trial Design

**Figure 1:**
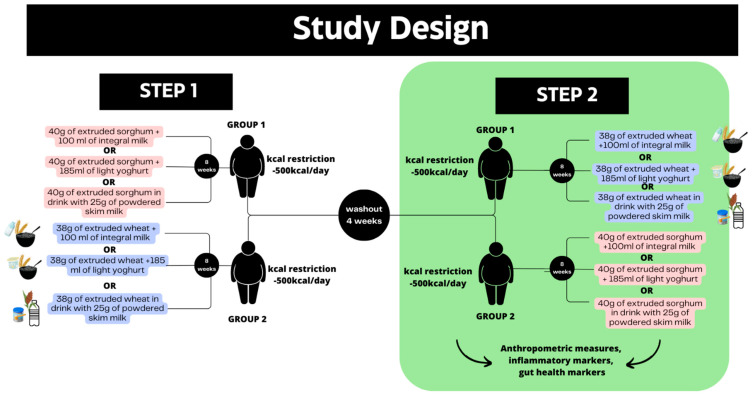
Study design and interventions. Data from the second phase of a crossover study were used in this study.

This was an 8-week, single-blind, controlled, randomized nutritional intervention study conducted in men with overweight. This study was conducted using data from the second phase of a crossover study previously conducted by our research group [29]. Samples related to intestinal health were collected only in the second phase of the study, pointing to the need for carrying out this investigative study. The previous study was a crossover, randomized, controlled, single-blind clinical trial, lasting 16 weeks and with 4 weeks washout period to eliminate residual effects of the first intervention period. The participants included in the study were randomly allocated in a 1:1 ratio to receive extruded SC319 whole sorghum or extruded whole wheat (Figure 1).

The study was approved by the Human Research Ethics Committee of the Federal University of Viçosa, Brazil (CAAE: 13630513.0.0000.5153). All participants were informed about the objectives of the study and provided written informed consent.

### 2.3. Participants

Volunteers were recruited in Viçosa-MG, Brazil, through advertisements on social networks, pamphlets, and posters. An email address and a telephone number were made available to individuals who were interested in participating in the study. In the first contact, the objectives and conditions of the study were informed to the potential volunteers. Then, screening was conducted for those who chose to participate in the study. During screening, a selection form was completed and, when they met the eligibility criteria, respecting the inclusion and non-inclusion criteria, a nutritional assessment was performed.

Eligibility criteria were male; age 18–40 years; body mass index (BMI) 27.0–34.9 kg/m^2^; waist circumference ≥ 90 cm; fasting capillary blood glucose 70–99 mg/dL; capillary cholesterol < 240 mg/dL; capillary triglycerides < 150 mg/dL; and absence of acute and chronic diseases other than obesity and food allergies. Non-inclusion criteria were individuals with eating disorders (lactose or gluten intolerances), the use of medications known to affect appetite, glycaemia, and energy or lipid metabolism; alcohol consumers and/or smokers; use of dietary fiber supplement; and recent changes in body weight (<5 kg in the past three months). Exclusion criteria were no consumption of the test food for more than six days (consecutive or not) and use of antibiotics during the intervention.

Only men were included in the study for two main reasons: the first is that males do not have as many hormonal changes as females, especially related to the menstrual period, considering that the woman’s reproductive status linked to the ovarian cycle is imperative while examining disparities between sexes in terms of health and vulnerability to diseases, scrutinizing the impacts of drugs, and exploring behavior. In addition, men suffer fewer changes in hormones associated with body composition compared to women, which meant that, in this case, male subjects were recruited for the study. The second reason is the fact that a previous experimental study investigated the consumption of sorghum in male Wistar rats fed a diet high in saturated fat (SFA) to induce overweight, since the consumption of this type of diet is associated with the development of obesity, so this study can be considered a sequel [23,24]. The individuals included in this study did not have diabetes, disorders in lipid metabolism, and/or arterial hypertension. The intervention time of 8 weeks was calculated by estimating a weight loss of 4 kg in two months, providing a weight loss of 3 to 5% of body weight, which is considered a beneficial effect for overweight and obese individuals [30].

### 2.4. Interventions and Test Meals

Volunteers attended the laboratory daily to consume the test preparations (breakfast cereal with milk or dairy product, or a drink) at breakfast and guided to follow a 500 kcal/day caloric restriction diet. On the weekends, the volunteers consumed the meals at home. The volunteers were instructed to consume the entire amount of test food provided. Thus, meals were offered to both groups in the form of breakfast cereal (extruded sorghum × wheat) added with whole milk or light whole yogurt, or a drink (extruded flours were mixed with skimmed milk powder, powdered juice, and sweetener) (Supplementary Figure S1). Variation between meal types (breakfast cereal/drink) was defined to increase adherence to the intervention. The amount of sorghum consumed daily was based on a usual portion of breakfast cereal (40 g) and on the volume of sorghum that the subjects could ingest in a meal based on previous tests [22]. The amount of extruded wheat (38 g) was adequate compared to the same nutritional composition of sorghum (40 g). Further, the meals had the same amount of milk or dairy products; however, for each meal the macronutrient content was different due to the ingredient composition. However, in each extruded sorghum or wheat meal a similar content of calories, macronutrients, and dietary fiber was offered [21]. All volunteers consumed milk, yogurt, or drink the same number of times. A dietitian professional calculated the caloric restriction (500 kcal/day) for both groups to achieve weight loss of 2 kg/month. At the beginning of the intervention, the individuals replied to a food frequency questionnaire (FFQ) to estimate the caloric consumption before the intervention. Then, they received a food prescription according to their individual energy and nutrient requirements, restricted to 500 kcal/day. The volunteers received a substitutive list of food, by food groups. During the intervention period, the adherence to the diet prescription was assessed using the food record of 3 non-consecutive days, including a weekend. They were guided to maintain the physical activity level. The dietary prescription was based on Dietary Reference Intakes (DRIs) [31], and the volunteers received a replacement food list organized by food groups.

### 2.5. Outcomes

The primary outcome of this study was the effect on anthropometric measurements such as body weight, waist circumference, sagittal abdominal diameter, waist-to-height ratio, and body fat percentage. The second outcome was the effect of the interventions on intestinal health, including short-chain fatty acid synthesis, fecal pH, gut microbiota composition, and inflammatory markers, such as interleukin 6, interleukin 10, and tumor necrosis factor-α.

### 2.6. Randomization, Allocation, and Sample Power

Participants were randomized using a random sequence, according to the corresponding numbers received prior to the intervention, using a Microsoft Excel 365 software spreadsheet for distribution between groups. Allocation concealment occurred so that investigators or research participants did not know whether the next eligible participant would receive a treatment or control intervention. This was masked until such time as the intervention was initiated.

A power of 94.54% was obtained considering the mean difference in body fat percentage between the groups (effect size = 1.64), bilateral α of 5%, and sample size of the groups. Calculations were performed using the GPower software version 3.1.9.7.

### 2.7. Assessment of Anthropometry and Body Composition Markers

Anthropometric and body composition evaluations were performed by a single trained researcher. Body weight was assessed using an electronic platform scale (Model 2096 PP, Toledo, Brazil), with a capacity of 150 kg and precision of 50 g. Height was measured using a stadiometer (Alturexata^®^, São Paulo, Brazil) fixed to the wall [32]. BMI (kg/m^2^) was computed and classified according to the WHO (2000). Waist circumference (WC) was measured to the nearest 0.1 cm with a flexible band at the midpoint between the last rib and the iliac crest (World Health Organization, 2000). WC ≥ 90 cm was adopted as a criterion to classify abdominal obesity. Sagittal abdominal diameter (SAD) was measured with a portable sliding beam abdominal caliper (Holtain Kahn Abdominal Caliper^®^, Holtain Ltd., Dyfed, Wales, UK) at the midpoint between the iliac crests. Waist-to-height ratio (WHtR) was calculated as WC divided by height (cm).

Body composition was assessed by dual-energy X-ray absorptiometry (DXA) (GE Healthcare, Lunar Prodigy Advance), and the results were expressed as total body fat (%). Body fat % higher than 25% was used to classify individuals with overweight [33].

### 2.8. Inflammatory Markers

All participants underwent overnight fasting, and blood samples were collected at baseline and endpoint. Enzyme-linked immunosorbent assay (ELISA) was utilized to analyze the levels of inflammatory markers, including interleukin 6, interleukin 10, and tumor necrosis factor-α. The Milliplex Map Human Cytokine/Chemokine Magnetic Bead kit (HCYTOMAG-code 60K, Millipore, Darmstadt, Germany) was employed for the accurate measurement of these markers in the blood samples.

### 2.9. Fecal Samples

At baseline and endpoint (8 weeks after) of the intervention, study participants were instructed to provide a fecal sample as close to the collection time as possible. If immediate processing was not feasible, the samples were refrigerated at 4 °C for a maximum of 12 h. Participants transported the fecal samples to the laboratory in polystyrene containers along with ice cubes to ensure temperature preservation. Upon arrival, the samples were weighed and transferred into micro tubes, then subsequently stored at −80 °C until analysis.

### 2.10. Fecal pH

The fecal pH level was assessed using a digital pH meter T-1000 (Tekna, São Paulo, Brazil). To perform the measurement, one gram of feces was transferred into a 15 mL falcon-type tube, and then 10 mL of ultrapure water was introduced. The mixture was thoroughly homogenized by vortexing, and the pH reading was obtained using a digital pH meter.

### 2.11. Organic Acid Analysis

To extract and identify organic acids from fecal samples, 500 mg of feces was weighed in duplicate and stored at −80 °C until further analysis. The frozen feces were thawed at room temperature (23 ± 2 °C) and homogenized with 1 mL of ultrapure water. Subsequently, the samples underwent centrifugation at 12,000× *g* for 10 min at 4 °C, using a Himac CT 15RE centrifuge (Hitachi, Tokyo, Japan). After centrifugation, the resulting supernatants were processed following the procedure described by Siegfried et al. (1984) [34]. High-performance liquid chromatography (HPLC) coupled with a refractive index (RI) Shodex RI-101 was employed to determine the levels of organic acids (acetic, succinic, formic, propionic, valeric, isovaleric, isobutyric, and butyric acid). The HPLC system used was Dionex Ultimate 3000 Dual detector HPLC (Dionex Corporation, Sunnyvale, CA, USA). A Bio-Rad HPX-87H column (300 mm × 4.6 mm) equipped with a Bio-Rad Cation H guard column was utilized under a column temperature of 45 °C, and the injection volume for each sample was 20 µL. The mobile phase for the chromatography was a mixture of concentrated sulfuric acid, EDTA, and ultrapure water, flowing at a rate of 0.7 mL/min. To generate the standard curve for quantification, the following organic acids were used at specific concentrations: acetic, succinic, formic, propionic, valeric, isovaleric, isobutyric, and butyric acid. The concentrations for the acids were set at 10 mmol/L, except for isovaleric acid at 5 mmol/L and acetic acid at 20 mmol/L.

### 2.12. Fecal Sample DNA Extraction

The extraction of DNA from fecal samples was performed using the QIAamp Fast DNA Stool Mini kit (Qiagen, Hilden, Germany) in accordance with the manufacturer’s guidelines. Each extraction involved 200 ± 20 mg of feces as the starting material. Following purification, the isolated DNA was preserved at −80 °C until it was ready for subsequent analysis.

### 2.13. Quantitative Real-Time Polymerase Chain Reaction (qPCR) Analysis of Gut Microbiota DNA Concentration

In this study, the researchers aimed to quantify the concentration of gut microbiota DNA through quantitative real-time polymerase chain reaction (qPCR) analysis. The DNA concentration was determined by measuring the absorbance at 260 nm (A260), while its purity was assessed by calculating the A260/A280 ratio using a Multiskan™ 1500 spectrophotometer (Thermo Fisher Scientifics; Waltham, MA, USA). For PCR analysis, group-specific primers (Supplementary Table S1) targeting the 16S rRNA gene were used. These primers were obtained from Alpha DNA e Diagnósticos Moleculares LTDA (Goiânia, GO, Brazil). The PCR reactions were performed using a CFX96 Touch™ Real-Time PCR Detection System (Bio-Rad, Hercules, CA, USA) with the Primer Express software version 3.0. Each well of the microtiter plate contained a reaction mixture consisting of QuantiNova SYBR^®^ Green PCR Kit (Qiagen, Hilden, Germany), forward and reverse primers at concentrations of 300 nM, and nuclease-free water, totaling 23 μL. To this mixture, 2.0 μL of each sample or standard was added, followed by brief centrifugation using a Labnet MPS1000 model, and then the plate was subjected to PCR analysis. The PCR amplification conditions included an initial denaturation of the DNA at 95 °C for 10 min, followed by 40 cycles of denaturation at 95 °C for 10 s, primer annealing at the optimized temperature for 20 s, and extension at 72 °C for 15 s. After amplification, a melting curve analysis was performed to distinguish the targeted PCR product from non-specific products. To determine the bacterial concentration in each sample, Ct (cycle threshold) values were obtained and compared to standard curves generated from serial dilutions of DNA isolated from pure cultures of different reference strains. These strains, including Bacteroides ovatus ATCC 8483, Escherichia coli ATCC 11775, and Lactobacillus delbrueckii UFV H2b20 CCT 3744, were obtained from the American Type Culture Collection (ATCC) and the Tropical Cultures Collection. The standard curves exhibited a linear relationship between cell numbers and Ct values (r^2^ = 0.99–0.96), allowing for accurate quantification of bacterial concentrations in the samples.

### 2.14. Analysis of Gut Microbiota

The sequencing of variable regions of the 16S rRNA gene of members of the Bacteria domains (V3–V4) was carried out by the company Argonne National Laboratory^®^ (Lemont, IL, USA) using the MiSeq platform (Illumina, San Diego, CA, USA). Data processing and analysis were performed using the Mothur v.1.40.0 program [35]. The sequences were aligned using the SILVA v.132 16S rRNA gene reference database [36]. The taxonomic classification was carried out using the same database mentioned above. The operational taxonomic unit (OTU) was grouped with a 97% similarity cutoff.

For alpha diversity analysis, the indices Chao1, Shannon, and Simpson were applied. Beta diversity was assessed by Principal Coordinate Analysis (PCoA) based on the Bray–Curtis dissimilarity index and a similarity test for non-parametric data (ANOSIM, permutation number = 1000) [37].

Metagenome functional predictive analysis was carried out using PICRUSt2 software version 2.3.0. Normalized OTU abundance was identified, and the assigned functional traits were predicted based on reference genomes using the Kyoto Encyclopedia of Genes and Genomes (KEGG). The most abundant metabolic processes and significant fold-change differences in functional pathways between groups adopting an unpaired *t*-test (to analyze sorghum at two points of data collection) or paired *t*-test (for beginning and endpoint group analysis) (α = 95%) using STAMP software version 2.1.3. were plotted.

### 2.15. Statistical Analysis

For body composition, inflammatory markers, organic acids, fecal pH, and PCR, statistical analysis was performed using SPSS 20.0 software. The normality of the data was assessed by Shapiro–Wilk test. The average of each variable at the beginning and end of the intervention was compared using paired *t*-test or Wilcoxon test. The difference between the averages of the variables at the beginning and end of the intervention periods were compared using Student’s *t*-test or the Mann–Whitney test. Averages and times were submitted to ANOVA followed by Newman–Keuls post hoc test or Kruskal–Wallis followed by Dunn’s post hoc test. Cohen’s d effect size was calculated from the difference between the means of the groups, divided by the mean of their SD. The magnitude of the effect was quantified as null (Cohen’s d < 0.19), small (Cohen’s d = 0.2 to 0.49), medium (Cohen’s d = 0.5 to 0.79), large (Cohen’s d = 0.8 to 1.29), and very large (Cohen’s d > 1.30) [38].

For analysis related to gut microbiota composition the normality of the data was assessed by Kolmogorov–Smirnov test. The α diversity index and Firmicutes/Bacteroidetes ratio statistical analysis was performed using GraphPad version 9.0. Principal Coordinate Analysis (PCoA) based on the Bray–Curtis dissimilarity index was accessed using Past software version 4.0.5. Metagenome functional predictive analysis was carried out using PICRUSt2 software version 2.3.0. Differences between averages were analyzed using STAMP software version 2.1.3. White’s non-parametric *t*-test was performed followed by Benjamin FDR correction. Statistically significant *p* values associated with microbial clades and functions identified by LEfSe were corrected by Benjamin FDR correction. The level of significance in two-tailed tests was set at 5%.

## 3. Results

**Figure 2:**
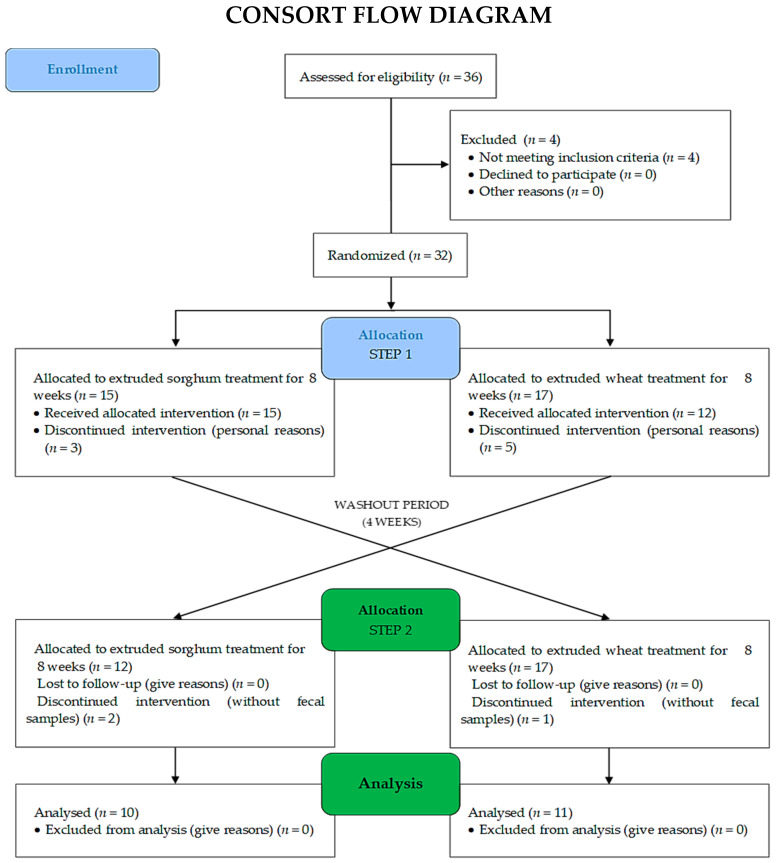
Study design of intervention protocol. The data utilized in this study were from step 2 of the intervention.

Thirty-six men with overweight were recruited to the study. However, 12 did not meet the inclusion criteria; therefore, a total of 24 participants were included in study. Three participants left the study for personal reasons and twenty-one individuals finished the study (Figure 2). The average age of participants in the second phase (*n* = 21) was 25.6 ± 4.6 years. At baseline, participants of both groups did not present differences in the anthropometric, biochemical, and food consumption markers.

### 3.1. Anthropometric Measures and Inflammatory Markers

Significant weight loss (−1.25 ± 0.84 kg, *p* = 0.0015), waist circumference (−2.33 ± 1.74 cm, *p* = 0.012), sagittal abdominal diameter (−0.70 ± 0.67 cm, *p* = 0.034), waist-to-height ratio (−0.011 ± 0.012, *p* = 0.047), and body fat percentage (−2.97 ± 1.91%, *p* = 0.013) reductions were observed after sorghum consumption for eight weeks compared to baseline (Table 1). The body fat percentage reduction was higher in the sorghum group when compared to the wheat group (−2.97 ± 1.91% vs. −0.16 ± 1.47%, *p* = 0.005), with a very large effect size (Cohen’s d = 1.67). Inflammatory markers were unchanged after sorghum consumption. However, wheat consumption increased IL-6 levels (Table 1).

### 3.2. SCFA Synthesis and Gut Microbiota PCR

We did not observe significant changes in fecal concentrations of acetic, propionic, and butyric acids after sorghum or wheat consumption for 8 weeks (Table 2). Despite this, a numeric increase in butyric acid was observed in the sorghum group compared to control, with a small effect size (Cohen’s = 0.35). In addition, fecal pH and intestinal microbiota composition did not change after 8 weeks of intervention (Table 2).

### 3.3. Gut Microbiota Analysis

Sequencing the 16S rRNA gene from stool samples generated 1,383,378 raw sequences. After filtering and cleaning the sequences, 1,001,142 good-quality sequences were obtained. The Good’s coverage obtained in samples was >99%, indicating good sequencing coverage. Raw filtered reads and normalized read counts per group are shown in Supplementary Table S2.

**Figure 3:**
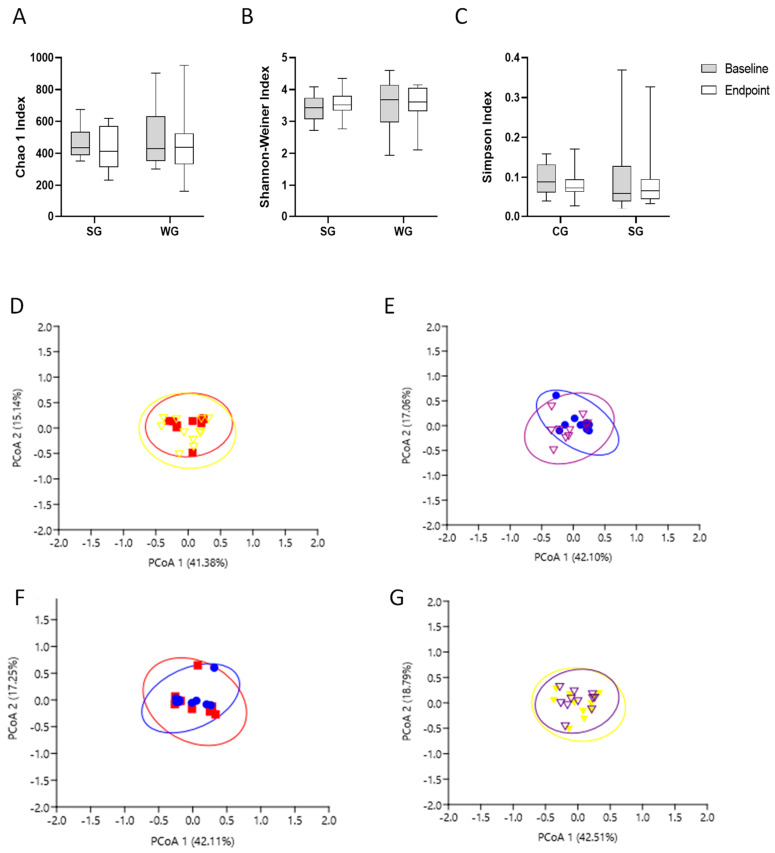
Alpha-diversity index at baseline and endpoint after 8-week intervention with test meals containing 40 g of extruded sorghum by day estimated by (**A**) Chao, (**B**) Shannon–Weiner, and (**C**) Simpson. Statistical analysis was performed using paired *t*-test or Wilcoxon test (sorghum and wheat group baseline vs. endpoint), or unpaired *t*-test or Wilcoxon test (sorghum × wheat group at baseline and endpoint). Significance was established as *p* < 0.05. Analyses were performed using Graphpad version 9.0. SG: sorghum group; WG: wheat group. Beta diversity was estimated by Principal Coordinate Analysis (PCoA) based on Bray–Curtis similarity distance of cecal microbial communities in men with obesity. The red squares presented sorghum group volunteers at baseline; yellow triangles presented sorghum group volunteers at endpoint. The blue circles presented wheat group volunteers at baseline and purple triangles presented wheat group volunteers at endpoint. (**D**) Sorghum intragroup; (**E**) wheat intragroup; (**F**) wheat and sorghum intergroups at baseline; (**G**) wheat and sorghum intergroups at endpoint. Permutational multivariate analysis of variance (PERMANOVA) was conducted in software STAMP version 2.0.2 considering α = 5%.

**Figure 4:**
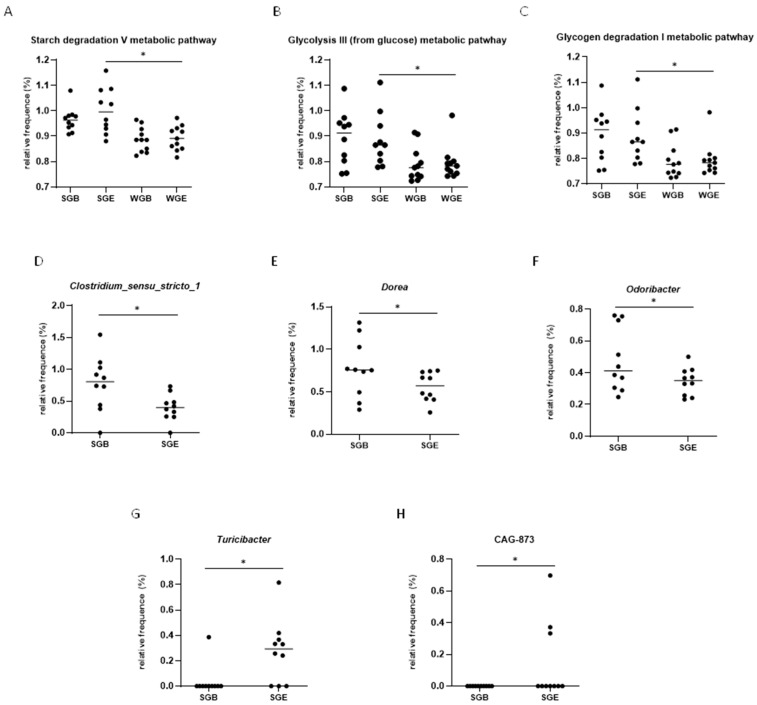
Microbial metabolic pathways in stool of men with obesity. Asterisks mean statistical difference between groups (*p* > 0.05). The statistical difference corresponds to the groups where the bar with an asterisk begins and ends. (**A**) starch degradation V metabolic pathway intergroup; (**B**) glycolysis III (from glucose) metabolic pathway intergroup; (**C**) glycogen degradation I metabolic pathway intergroup in stool of men with overweight at endpoint by White’s parametric *t*-test; (**D**) *Clostridium_sensu_stricto_1* sorghum intragroup relative abundance; (**E**) *Dorea* sorghum intragroup relative abundance; (**F**) *Odoribacter* sorghum intragroup relative abundance; (**G**) *Turicibacter* sorghum intragroup relative abundance; (**H**) CAG-873 sorghum intragroup relative abundance. Statistical analysis was performed in STAMP software version 2.1.3. considering α = 0.05. SGB: sorghum group at baseline; SGE: sorghum group at endpoint; WGB: wheat group at baseline; WGE: wheat group at endpoint.

The α diversity, microbial richness, and diversity were not different for the Chao1 (Figure 3A), Simpson (Figure 3B), and Shannon (Figure 3C) indices at baseline and the endpoint for intra- and intergroups. The β diversity intragroup compared to the sorghum group represented approximately 59.36% (Figure 3D) and compared to the wheat group represented approximately 61.30% of the dissimilarity in bacterial species composition (Figure 3E), according to the Principal Coordinate Analysis (PCoA). The β diversity intergroup at baseline represented approximately 56.52% (Figure 3F) and at the endpoint represented 59.16% of the dissimilarity in bacterial species composition (Figure 3G), according to the PCoA. The clustering of the bacterial community did not present intergroup differences at the phyla, class, order, family, or genera level. Furthermore, a reduction in Clostridium_sensu_strictu1, Dorea, and Odoribacter (Figure 4D–F) and an increase in CAG-873 and Turicibacter (Figure 4G,H) at the endpoint were observed in the sorghum intragroup.

Intervention groups presented 21 phyla, 35 classes, 77 orders, 134 families, and 302 genera. All groups had eight predominant phylum: Firmicutes (sorghum group: 61.45 ± 4.92%; wheat group: 62.81 ± 7.55%), followed by Bacteroidetes (sorghum group: 29.35 ± 4.67%; wheat group: 26.17 ± 6.92%), Proteobacteria (sorghum group: 3.87 ± 1.09%; wheat group: 4.34 ± 2.18%), Actinobacteria (sorghum group: 2.75 ± 1.18%; wheat group: 3.10 ± 1.02%), and Desulfobacterium (sorghum group: 1.04 ± 0.47%; wheat group: 1.06 ± 0.42%) (Supplementary Figure S2A,B). The intra- and intergroup Firmicutes/Bacteroidetes ratio was similar (*p* > 0.05) (Supplementary Figure S2C).

### 3.4. Functional Prediction Analysis (KEGG Analysis)

According to the intragroup KEGG metabolic pathway analysis, extruded sorghum SC319 consumption increased methyl ketone biosynthesis (*p* = 0.04) and protein N-glycosylation (bacterial) (*p* = 0.01), while it decreased GDP-D-glycero-α;-D-manno-heptose biosynthesis (*p* = 0.01), adenosine nucleotides degradation II (*p* = 0.02), guanosine nucleotides degradation III (*p* = 0.02), and fucose degradation (*p* = 0.04) metabolic pathways (Supplementary Figure S3A).

By comparing the extruded sorghum and wheat group metabolic pathways using a KEGG intergroup analysis at the endpoint, it was found that sorghum consumption increased S-adenosyl-L-methionine cycle I (*p* < 0.001), the NAD salvage pathway I (*p* < 0.001), N10-formyl-tetrahydrofolate biosynthesis (*p* < 0.001), starch degradation V (*p* < 0.001) (Figure 4A), the pentose phosphate pathway (non-oxidative branch) (*p* = 0.003), L-lysine biosynthesis VI (*p* = 0.005), glycolysis III (from glucose) (*p* = 0.006) (Figure 4B), glycogen degradation I (bacterial) (*p* = 0.007) (Figure 4C), colonic acid building blocks biosynthesis (*p* = 0.012), NAD biosynthesis I (from aspartate) (*p* = 0.013), adenosylcobalamin biosynthesis from cobyrinate a,c-diamide I (*p* = 0.017), adenosylcobalamin salvage from cobinamide II (*p* = 0.021), adenosylcobalamin salvage from cobinamide I (*p* = 0.038), the superpathway of sulfur oxidation (*Acidianus ambivalens*) (*p* = 0.043), L-isoleucine biosynthesis III (*p* = 0.045), flavin biosynthesis I (bacteria and plants) (*p* = 0.047), reduced L-methionine biosynthesis I (*p* = 0.003), the superpathway of S-adenosyl-L-methionine biosynthesis (*p* = 0.003), the superpathway of L-methionine biosynthesis (trans-sulfuration) (*p* = 0.004), the aspartate superpathway (*p =* 0.006), ADP-L-glycero-β;-D-manno-heptose biosynthesis (*p* = 0.012), tRNA processing (*p =* 0.016), pyrimidine deoxyribonucleotides de novo biosynthesis III (*p* = 0.021), the superpathway of L-lysine, L-threonine and L-methionine biosynthesis I (*p* = 0.023), the superpathway of heme biosynthesis from uroporphyrinogen-III (*p =* 0.024), heme biosynthesis II (anaerobic) (*p =* 0.034), the superpathway of glycerol degradation to 1,3-propanediol (*p =* 0.047), and the superpathway of polyamine biosynthesis I (*p =* 0.048) compared to wheat consumption (Supplementary Figure S3B).

### 3.5. LEfSe Analysis

All OTUs were analyzed by a Linear Discriminant Analysis of Effect Size (LEfSe) to identify the dominant cecal microbiota and intestinal biomarkers using taxonomy (Supplementary Figure S4A). As a result, four dominant OTUs with an effect size > 3 were identified. The sorghum and wheat groups did not show a higher number of dominant taxa in the intergroup at baseline and endpoint. For the intragroup, sorghum showed a higher effect size on the dominant community of Butyrucunibas (Supplementary Figure S4B) and Turicibacter (Supplementary Figure S4C) at the endpoint, and at baseline to UCG_002 (Supplementary Figure S4D) and UBA1819 (Supplementary Figure S4E).

## 4. Discussion

This study investigated the effects of the consumption of extruded SC319 whole sorghum or extruded whole wheat associated with an 8-week daily 500 kcal energy restriction diet on the modulation of intestinal health with a focus on gut microbiota, short-chain fatty acid production, fecal pH, and weight loss and inflammation markers. Extruded sorghum and whole wheat did not show differences in α and β diversity indexes, short-chain fatty acid synthesis, and fecal pH. However, sorghum consumption promoted alterations at the genus level compared to baseline, reducing *Clostridium_sensu_stricto 1*, *Dorea*, and *Odoribacter* and increasing CAG-873 and *Turicibacter*. The sorghum group showed a lower body fat percentage than the whole wheat group at the endpoint. These effects are likely due to sorghum’s chemical composition of 3-deoxyanthocyanins, resistant starch, and proanthocyanidins [18].

Extruded whole wheat and sorghum have a similar concentration of total dietary fiber, promoting a similar dietary fiber intake when consumed for eight weeks. This resulted in similar effects on fecal pH, short-chain fatty acid synthesis, and microbial diversity. However, sorghum SC319 promoted a weight loss intragroup, probably due to its unique phenolic compounds, 3-deoxyanthocianins, and proanthocyanins, which complex with starch, thereby reducing their digestibility [27] and making them available to be fermented by the intestinal microbiota, favoring weight loss. These effects were confirmed in our study by a KEGG analysis, in which the sorghum group activated starch and glycogen degradation pathways compared to wheat.

The extruded SC319 sorghum consumption tended to reduce the proteobacteria phylum, most of which is pathogenic. At the genus level, *Clostridium sensu stricto 1*, *Dorea,* and *Odoribacter* were reduced at the endpoint. *Clostridium sensu stricto 1* is a cluster of *Clostridium* species that includes commensal and pathogenic species. Members of this cluster exhibit a consistent capacity to synthesize butyrate [39], which may not have altered butyrate synthesis in the sorghum group. *Dorea* is a microorganism positively associated with prediabetes and glucose concentrations [40]. Although the individuals did not present a change in glucose metabolism in our study, the reduction in *Dorea* can be considered a protective effect for hyperglycemia, insulin resistance, and other disturbances in glucose metabolism, resulting in diabetes mellitus. *Odoribacter* was correlated with the expression of inflammatory cytokines such as TNFα and IFNγ [41]. However, no difference was observed in TNFα serum concentrations in our study. Furthermore, extruded SC319 sorghum consumption increased *Turicibacter* and CAG_873 at the genus level. One study showed that *Turicibacter* is correlated with the pro-inflammatory cytokine IL-1 [42], and another pointed to overall immune activation [43]; however, we did not observe differences in inflammation markers.

We observed that overweight male individuals intaking 40 g/day of extruded whole sorghum SC319, rich in 3-deoxyanthocyanidins and proanthocyanidins, for eight weeks, did not change the anti-inflammatory markers. A previous study with these individuals also showed no effects on the antioxidant response [29]. On the other hand, the overweight male individuals intaking 38 g/day of extruded whole wheat had increased IL-6 concentrations. The wheat allergenicity likely promoted this effect because the sensitization to gluten can moderate antigen-specific inflammatory markers such as IL-6 [44]. Further, we did not observe modulation in microbiota composition, which did not change inter- and intragroup SCFAs in the wheat group.

The intake of extruded sorghum is an alternative to wheat consumption, which can improve the life quality of populations, especially individuals with celiac disease. Thus, sorghum is an excellent substitute for wheat intake since it does not have allergen compounds and, when combined with other ingredients, can be used to produce beverages, pasta, bread, cakes, and biscuits, among other bakery products. The unique techno-functional and biofunctional properties of kafirins, such as non-allergenic features and slow digestibility by mammalian proteases, amplify the applications of sorghum flour in food production [45]. Furthermore, the phenolic compounds present in sorghum grains may exert other beneficial effects on the body, such as improving non-communicable chronic diseases, glucose metabolism, and adipogenesis [23,25,46].

Although no effects were observed regarding the composition of the intestinal microbiota in the extruded whole sorghum and wheat groups, sorghum can be an alternative to wheat since it is a non-allergenic cereal and has been demonstrated to enhance weight loss, in addition to showing similar results to wheat on SCFA synthesis and fecal pH.

The strength of the present study is to associate two interventions, namely the use of extruded whole sorghum, a prebiotic, with a caloric restriction diet, to investigate the systemic effects on body composition, inflammatory markers, and intestinal health, encouraging the consumption of this cereal in the human population. The limitations of this study are as follows: the small number of volunteers in each group; the unusual consumption of sorghum by most of the world population, besides being considered a whole ingredient by Brazilian Official Institutions; the duration of the intervention, because eight weeks may not be a sufficient amount of time to find effects on intestinal health; gut microbiota composition; and the data collection, due to it being in the second phase of a crossover study, as weight loss is more effective in the first weeks with a caloric restriction diet [47]. In addition, future studies need to consider the increase in sorghum intake, because cereals constitute a significant component of the diet, and we can use sorghum to prepare other food products. Thus, our study revealed that the daily consumption of 40 g of extruded sorghum SC319 for eight weeks enhances weight loss, likely due to the phenolic compounds, 3-deoxyanthocianins, and proanthocyanidins. Furthermore, extruded whole sorghum has a similar effect as extruded whole wheat on intestinal health, with no differences in intestinal microbiota, SCFA synthesis, and fecal pH.

## 5. Conclusions

Consuming SC319 extruded sorghum allied to an energy restriction diet reduced body fat percentage in Brazilian men with overweight compared to control, with no differences in SCFA synthesis, fecal pH, α and β diversity, and inflammatory markers. In addition to this, considering gut microbiota functional prediction, the extruded SC319 consumption for eight weeks improved metabolic pathways related to carbohydrate metabolism compared to extruded wheat consumption. The sorghum consumption intragroup had improved weight loss, decreased anthropometric measures, and a relative abundance of harmful microorganisms at the genus level.

## Figures and Tables

**Table 1:** Anthropometric variables and inflammatory markers serum concentrations of the participants at baseline and endpoint by treatment.

Variables	Sorghum Group (*n* = 12)		Wheat Group (*n* = 10)		Δ *p* Value ^1^	Cohen’s d (95% CI)
	Baseline	Endpoint	Baseline	Endpoint		
Weight (kg)	84.37 ± 7.09 ^a^	83.12 ± 6.96 ^b^	91.83 ± 11.49 ^a^	90.68 ± 11.10 ^a^	0.919	−0.06 (−0.9; 0.78)
BMI (kg/m^2^)	28.16 ± 0.96 ^a^	27.14 ± 0.98 ^b^	28.83 ± 2.10 ^a^	28.49 ± 2.38 ^a^	0.907	−0.16 (−1.00; 0.68)
WC (cm)	94.46 ± 3.47 ^a^	92.18 ± 3.84 ^b^	100.60 ± 6.10 ^a^	99.59 ± 6.79 ^a^	0.169	−0.66 (−1.52; 0.20)
SAD (cm)	20.83 ± 1.20 ^a^	20.13 ± 0.99 ^b^	22.64 ± 1.98 ^a^	22.11 ± 1.46 ^a^	0.673	−0.10 (−0.94; 0.74)
WHtR	0.54 ± 0.04 ^a^	0.53 ± 0.01 ^b^	0.57 ± 0.04 ^a^	0.56 ± 0.05 ^a^	0.346	−0.37 (−1.21; 0.48)
BF (%)	29.15 ± 4.53 ^a^	26.18 ± 4.97 ^b^	31.77 ± 6.68 ^a^	31.61 ± 6.91 ^a^	0.005	−1.67 (−2.64; −0.7)
**Inflammatory Markers**	**Baseline**	**Endpoint**	**Baseline**	**Endpoint**	**Δ *p* Value ^1^**	
IL-6 (pg/mL)	0.87 ± 0.19 ^a^	1.15 ± 0.03 ^a^	0.77 ± 0.28 ^a^	1.01 ± 0.18 ^b^	0.703	−0.21 (−1.05; 0.64)
IL-10 (pg/mL)	1.50 ± 1.01 ^a^	1.73 ± 0.88 ^a^	0.96 ± 0.49 ^a^	1.17 ± 0.62 ^a^	0.848	−0.09 (−0.93; 0.75)
TNFα (pg/mL)	7.54 ± 5.10 ^a^	9.02 ± 5.34 ^a^	6.41 ± 2.60 ^a^	7.02 ± 2.74 ^a^	0.274	0.58 (−0.28; 1.44)

*n* = 11 participants in each group. Data are expressed as mean ± standard deviation. BMI: body mass index, WC: waist circumference, SAD: sagittal abdominal diameter, WHtR: waist-to-height ratio, BF: body fat percentage, IL-6: interleukin 6, IL-10: interleukin 10; TNFα: tumor necrosis factor alpha. Different letters on the same line for each group mean *p* < 0.05 from paired *t*-test or for Wilcoxon matched-pairs signed-rank test, as statistical within-group differences (baseline vs. endpoint). ^1^ *p* < 0.05 from Student’s *t*-test or for Mann–Whitney test, as statistical significance between diet differences (sorghum vs. wheat).

**Table 2:** Effect of sorghum and wheat consumption on concentration of volatile fatty acids (VFAs) of overweight subjects and percentages of target species in samples relative to total bacteria content.

Variables	Sorghum Group		Wheat Group		Δ *p* Value between Groups ^2^	Cohen’s d (95% CI)
	Baseline (*n* = 10)	Endpoint (*n* = 10)	Baseline (*n* = 11)	Endpoint (*n* = 11)		
Total VFAs ^1^	28.35 ± 11.83 ^a^	31.82 ± 11.78 ^a^	29.65 ± 10.41 ^a^	27.42 ± 12.87 ^a^	0.436	0.06 (−0.78; 0.9)
Acetic acid	14.73 ± 1.96 ^a^	14.76 ± 3.7 ^a^	14.09 ± 3.52 ^a^	14.56 ± 5.40 ^a^	0.809	0.20 (−0.65; 1.04)
Propionic acid	6.32 ± 2.7 ^a^	6.05 ± 3.12 ^a^	7.14 ± 3.99 ^a^	6.18. ± 2.90 ^a^	0.641	−0.06 (−0.9; 0.78)
Butyric acid	4.90 ± 2.51 ^a^	5.46 ± 3.77 ^a^	5.89 ± 2.99 ^a^	5.01 ± 2.90 ^a^	0.456	0.35 (−0.5; 1.19)
Fecal pH	6.73 ± 0.37 ^a^	6.68 ± 0.55 ^a^	6.85 ± 0.45 ^a^	6.96 ± 0.53 ^a^	0.254	-
**Target taxon**	**Baseline ** ** (*n* = 10)**	**Endpoint ** ** (*n* = 10)**	**Baseline ** ** (*n* = 11)**	**Endpoint ** ** (*n* = 11)**	**Δ *p* Value between Groups ^1^**	
Bacteroidetes	108.89 ± 40.00 ^a^	106.51 ± 24.66 ^a^	73.96 ± 51.99 ^a^	106.39 ± 53.13 ^a^	0.115	-
Proteobacteria	0.19 ± 0.25 ^a^	0.20 ± 0.21 ^a^	1.30 ± 1.67 ^a^	3.56 ± 11.00 ^a^	0.075	-
Firmicutes	4.16 ± 1.53 ^a^	6.29 ± 4.54 ^a^	10.74 ± 11.30 ^a^	4.74 ± 2.97 ^a^	0.250	-

^1^ Total VFAs (mmol/L), acetic acid, propionic acid, butyric acid, isobutyric acid, formic acid, succinic acid, valeric acid, and isovaleric acid (mol/100 mol). Different letters on the same line for each group mean *p* < 0.05 from paired *t*-test or for Wilcoxon matched-pairs signed-rank test, as statistical within-group differences (baseline vs. endpoint). ^2^ *p* < 0.05 from Student’s *t*-test or for Mann–Whitney test, as statistical significance between diet differences (sorghum vs. wheat).

## Data Availability

Data presented in this study are available upon request to the corresponding author. The data are not publicly available due to the fact that they are available within an internal database of the research institution, therefore, they cannot be made publicly available.

## Supplementary Materials

The following supporting information can be downloaded at: https://www.mdpi.com/article/10.3390/nu15173786/s1, Figure S1. Details of the meals consumed throughout the study; Figure S2. Relative abundance at phylum level at the end of intervention with consumption of extruded sorghum SC319 during 8 weeks in the cecal microbiota. (A) Bacterial composition at phylum level of sorghum group (*n* = 10); (B) bacterial composition at phylum level of wheat group (*n* = 11); (C) Firmicutes to Bacteroidetes ratio at baseline and endpoint of sorghum and wheat groups. Data of cecal microbiota were analyzed by Dunn’s test with FDR and Bonferroni corrections in software STAMP version 2.0.2, considering a significance of *p* < 0.05. Firmicutes to Bacteroidetes ratio was analyzed using paired *t*-test or Wilcoxon test (sorghum and wheat groups baseline vs. endpoint), or unpaired *t*-test or Wilcoxon test (sorghum x wheat group at baseline and endpoint). Significance was established as *p* < 0.05. Analyses were performed using Graphpad version 9.0. SG: sorghum group; WG: wheat group.; Figure S3. Microbial metabolic pathways in stool of man with obesity. (A) Microbial metabolic pathways in stool of man with obesity that received a meal containing 40 g of extruded sorghum for 8 weeks at baseline and endpoint by White’s parametric *t*-test. (B) Microbial metabolic pathways in stool of man with obesity that received a meal containing 38 g of extruded wheat for 8 weeks at baseline and endpoint by White’s parametric *t*-test. All metabolic pathways that showed differences were appointed in the figures. Statistical analyses were performed in STAMP software considering α = 0.05. SB: sorghum group at baseline; SE: sorghum group at endpoint; WB: wheat group at baseline; WE: wheat group at endpoint.; Figure S4. Histogram of LEfSe method to compute linear discriminant analysis (LDA) scores of differences in dominant microorganisms between baseline and endpoint of sorghum group. SGB: sorghum group at baseline; SGE: sorghum group at endpoint. Significant differences were considered with *p* < 0.05.; Table S1. PCR primer sequences used; Table S2. Sequencing data at baseline and at the end of 8 weeks of interventions, according to each group.
