# Supplementary material for: Consumption of Extruded Sorghum SC319 Improved Gut Microbiota at Genus Level and Reduced Anthropometric Markers in Men with Overweight: A Randomized Controlled Clinical Trial
Source: Nutrients. 2023 Aug 30;15(17):3786. doi: 10.3390/nu15173786 (PMC10490362; doi:10.3390/nu15173786)
Supplement: Supplementary file 1 [file nutrients-15-03786-s001.zip › nutrients-2531716-supplementary.pdf]

nutrients

MDPI

Supplementary Materials

Consumption of Extruded Sorghum SC319 Improved Gut Microbiota at Level Genus and Reduced Anthropometric Markers in Men with Overweight: A Randomized Controlled Clinical Trial

Haira Lúcio [1,†], Pâmela Anunciação [1,†], Barbara da Silva [1], Alessandra da Silva [1], Valéria Queiroz [2], Carlos de Carvalho [3], Helena Pinheiro-Sant'Ana [1] and Hercia Martino [1,*]

Table S1. PCR primers sequences used.

Group	Primer sequences	Standard genomic DNA	References
**Total Bacteria**	F- GCAGGCCTAACACATGCAAGTC<br>R- CTGCTGCCTCCCGTAGGAGT	*Escherichia coli*	(Castillo et al., 2006)
**Firmicutes**	F- ATGTGGTTTAATTCGAAGCA<br>R- AGCTGACGACAACCATGCAC	*Lactobacillus delbrueckii*	(Guo et al., 2008)
**Bacteroidetes**	F- CATGTGGTTTAATTCGATGAT<br>R- AGCTGACGACAACCATGCAG	*Bacteroides ovatus*	(Guo et al., 2008)
**Proteobacteria**	F- CATGACGTTACCCGCAGAAGAAG<br>R- CTCTACGAGACTCAAGCTTGC	*Escherichia coli*	(Friswell et al., 2010)

Oligonucleotides used as primers (F: forward; R: reverse) for quantification of 16S rDNA genes.

Table S2. Sequencing data at baseline and at the end of 8 weeks of interventions, according to each group.

**Treatment**		**Good's coverage**	**Raw Sequences**	**After filtering and cleaning**		**After normalization**	
			**Reads**	**Reads**	**OTUs**	**Reads**	**OTUs**
**WG**	B	0.997 ± 0.001	34755 ± 11648	25255 ± 8518	283 ± 60	12783 ± 39	315 ± 101
	E	0.997 ± 0.001	32591 ± 13435	23709 ± 9609	326 ± 74	12792 ± 19	290 ± 93
**SG**	B	0.997 ± 0.001	29315 ± 5997	21249 ± 3868	341 ± 116	12792 ± 22	274 ± 45
	E	0.997 ± 0.001	34942 ± 6300	25004 ± 3966	314 ± 115	12779 ± 33	292 ± 79

Values presented in mean ± standard deviation. WG: wheat group; SG: sorghum group; B: baseline; E: endpoint.

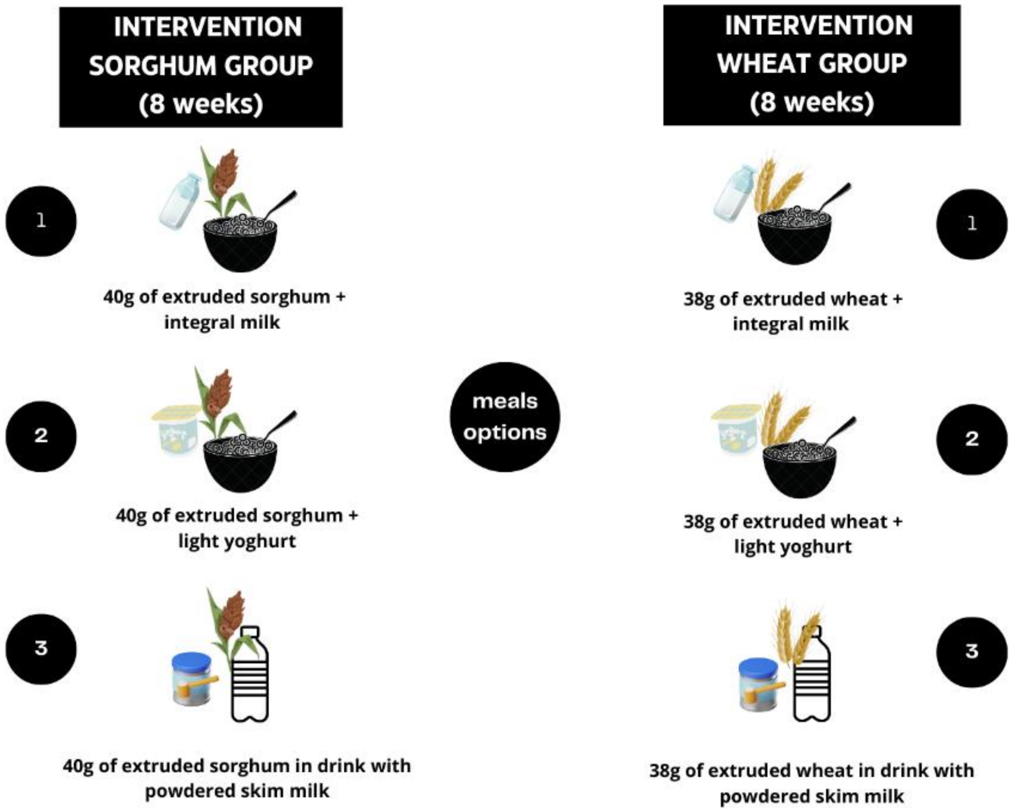

Figure S1. Details of the meals consumed throughout the study.

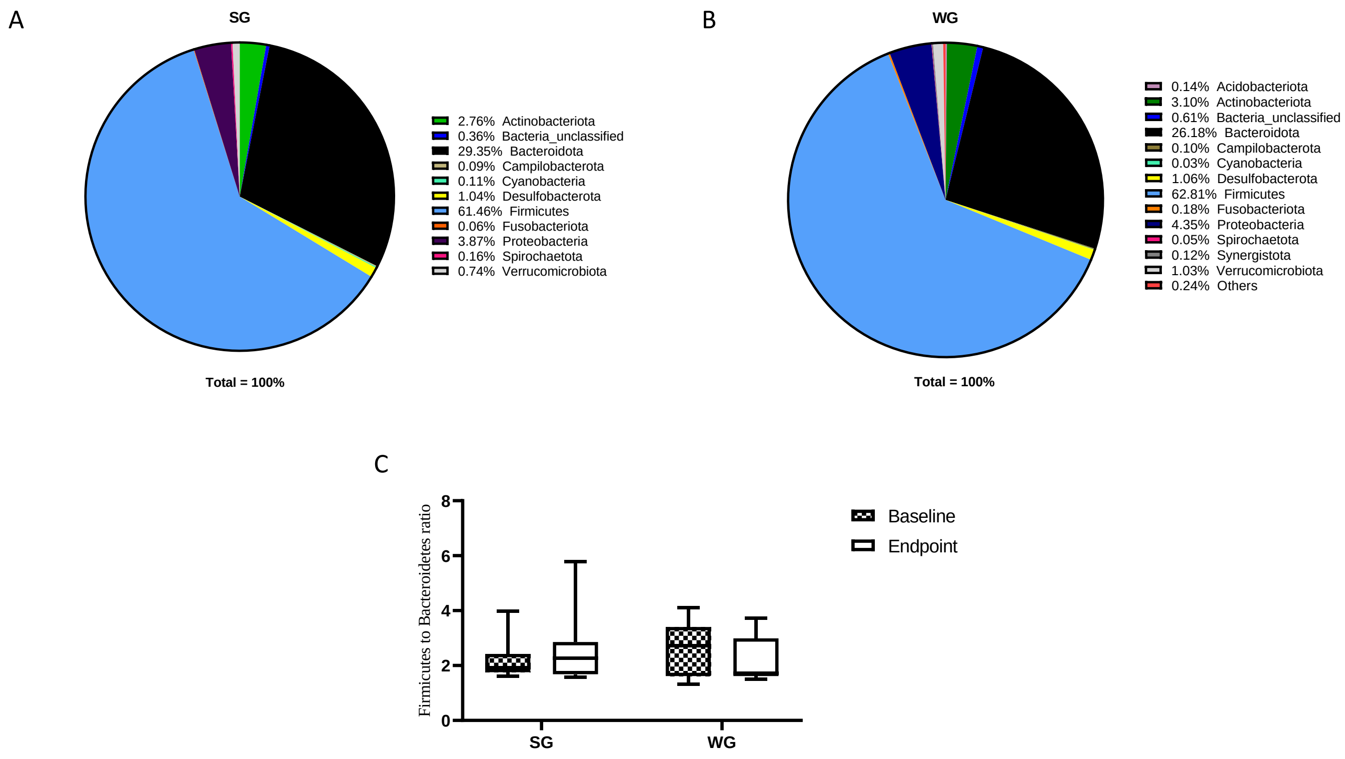

Figure S2. Relative abundance at phylum level at the end of intervention with consumption of extruded sorghum SC319 during 8 weeks in the cecal microbiota. (A) Bacterial composition at phylum level of sorghum group (n=10); (B) Bacterial composition at phylum level of wheat group (n=11); (C) Firmicutes to Bacteroidetes ratio at baseline and endpoint of sorghum and wheat groups. Data of cecal microbiota were analyzed by Dunn's test with FDR and Bonferroni corrections in software STAMP version 2.0.2, considering a significance of $p < 0.05$. Firmicutes to Bacteroidetes ratio was analyzed using paired t-test or Wilcoxon test (sorghum and wheat groups baseline vs endpoint), or unpaired t-test or Wilcoxon test (sorghum x wheat group at baseline and endpoint). Significance was established $p < 0.05$. Analyzes were performed using Graphpad version 9.0. SG: sorghum group; WG: wheat group.

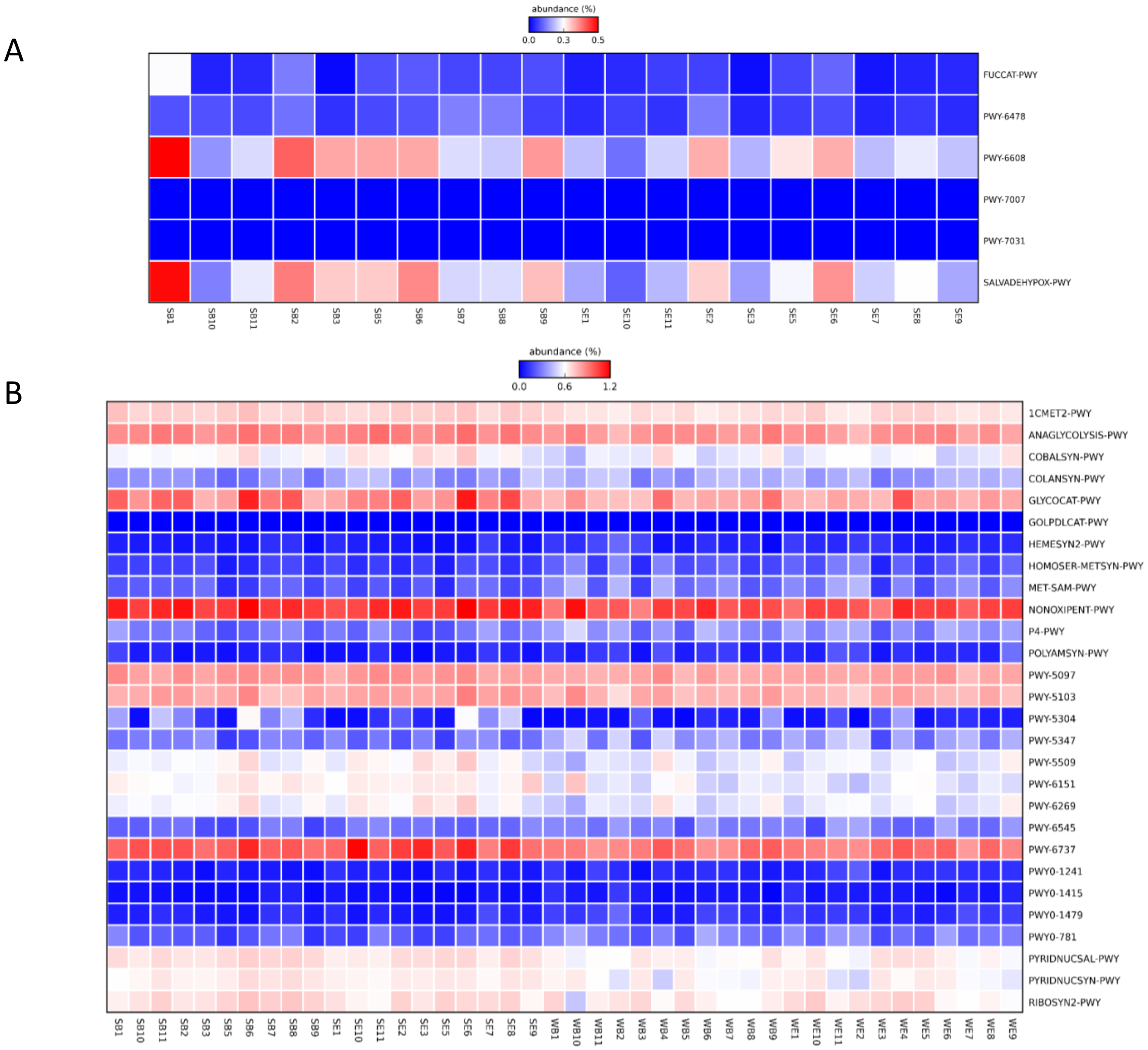

Figure S3. Microbial metabolic pathways in stool of man with obesity. (A) Microbial metabolic pathways in stool of man with obesity that received a meal containing 40g of extruded sorghum for 8 weeks at baseline and endpoint weeks by White's parametric t test. (B) Microbial metabolic pathways in stool of man with obesity that received a meal containing 38g of extruded wheat for 8 weeks at baseline and endpoint weeks by White's parametric t test. All metabolic pathways that showed differences were appointed in the figures. Statistical analyzes were performed in STAMP software considering α=0.05. SB: sorghum group at baseline; SE: sorghum group at endpoint; WB: wheat group at baseline; WE: wheat group at endpoint.

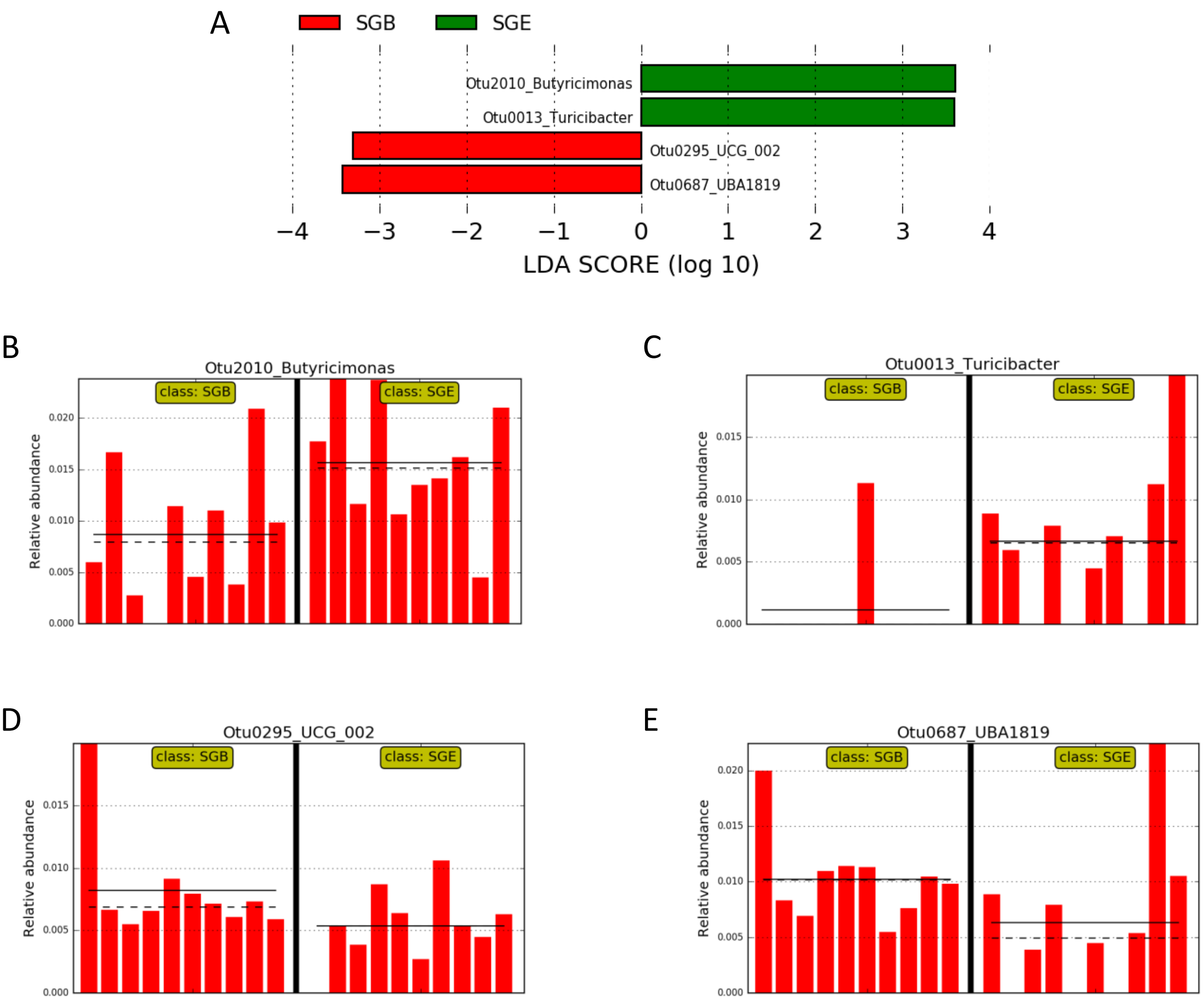

Figure S4. Histogram of LEfSe method to compute linear discriminant analysis (LDA) scores of differences in dominant microorganisms between baseline and endpoint of sorghum group. SGB: sorghum group at baseline; SGE: sorghum group at endpoint. Significant differences were considered with p<0.05.
